# CLEAR-HPV: Interpretable concept discovery for human-papillomavirus-associated morphology in whole-slide histology

**DOI:** 10.1016/j.patter.2026.101588

**Published:** 2026-06-16

**Authors:** Weiyi Qin, Yingci Liu-Swetz, Shiwei Tan, Hao Wang

**Affiliations:** 1 Department of Computer Science, Rutgers University, New Brunswick, NJ, USA; 2 Rutgers Health, Rutgers University, Newark, NJ, USA

**Keywords:** computational pathology, multiple instance learning, interpretable deep learning, concept discovery, head and neck cancer, human papillomavirus, HPV, whole-slide image analysis

## Abstract

Human papillomavirus (HPV) status is a critical determinant of prognosis and treatment response in head and neck and cervical cancers. Although attention-based multiple instance learning (MIL) achieves strong slide-level prediction for HPV-related whole-slide histopathology, it provides limited morphologic interpretability. To address this limitation, we introduce concept-level explainable attention-guided representation for HPV (CLEAR-HPV), a framework that restructures the MIL latent space to enable concept discovery without requiring concept labels during training. Within an attention-weighted latent space, CLEAR-HPV automatically discovers keratinizing, basaloid, and stromal morphologic concepts; generates spatial concept maps; and represents each slide with a compact concept-fraction vector. Its concept-fraction vectors preserve the predictive information of the original MIL embeddings while reducing the high-dimensional feature space (e.g., 1,536 dimensions) to only 10 interpretable concepts. CLEAR-HPV demonstrates consistent concept structure across The Cancer Genome Atlas (TCGA)-HNSCC, TCGA-CESC, and Clinical Proteomic Tumor Analysis Consortium (CPTAC) -HNSCC, providing compact, concept-level interpretability through a general, backbone-agnostic framework for attention-based MIL models of whole-slide histopathology.

## Highlights

• Discovers interpretable histology concepts without manual labels
• Links morphology patterns to HPV status in pathology images
• Preserves accuracy while reducing features to compact concepts
• Generalizes across datasets and staining conditions

## The bigger picture

Artificial intelligence is increasingly used to analyze pathology images and predict clinically important outcomes, such as viral status in cancers, including head and neck and cervical cancer, where human papillomavirus (HPV) status has major prognostic significance. Most current models, however, act as “black boxes,” offering little insight into what visual patterns they rely on. This limits their clinical trustworthiness and scientific value, as pathologists cannot easily verify or learn from their decisions.

Here, we present a framework that enables interpretable analysis of whole-slide histology images by identifying meaningful visual patterns directly from data without requiring manual annotation. Methods like this one, which make complex image-based models more transparent, provide a step toward AI systems that not only predict outcomes but also help explain disease biology. Such interpretable frameworks could improve clinical adoption of AI and support new discoveries in pathology by revealing how visual tissue patterns relate to disease mechanisms.

## Graphical abstract

**Figure:**
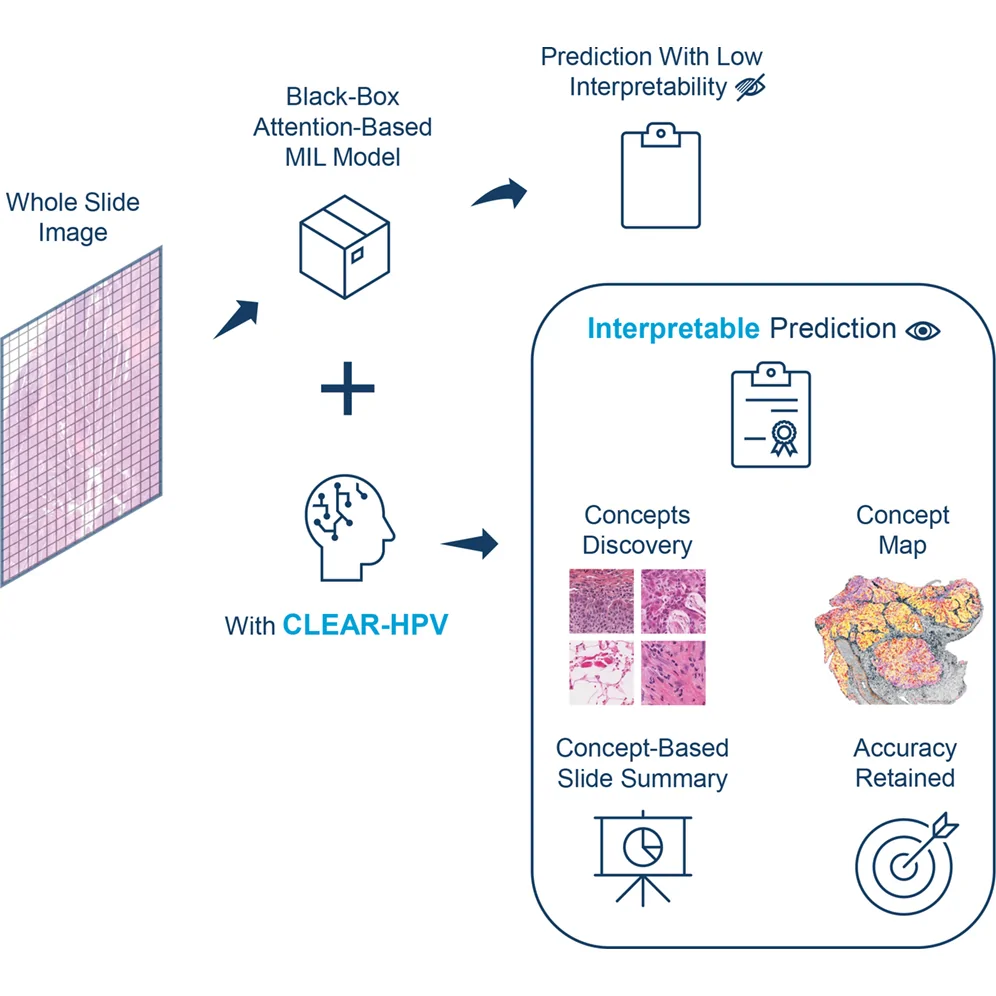

## Teaser

Understanding how AI models interpret pathology images remains a major challenge. Here, the authors introduce CLEAR-HPV, an interpretable framework that identifies meaningful tissue patterns linked to human papillomavirus status directly from whole-slide images without manual annotation. By transforming complex model representations into compact, biologically meaningful concepts, the approach maintains predictive accuracy while improving transparency. This work highlights how interpretable AI can bridge prediction and biological understanding in computational pathology.

## Introduction

Human papillomavirus (HPV)-associated head and neck and cervical cancers together account for 690,000 new cases worldwide each year,^1^ with HPV status strongly stratifying survival, treatment intensity, and long-term functional outcomes, making accurate and interpretable HPV assessment a problem of major clinical and public health significance. HPV-positive tumors are often non-keratinizing or basaloid,^2^^,^^3^^,^^4^ whereas HPV-negative tumors more commonly display keratinizing squamous morphology.^2^^,^^3^^,^^5^ However, substantial morphologic overlap and variability mean that HPV status cannot be reliably inferred by human observers from routine histologic assessment alone^6^; ancillary immunohistochemical or molecular assays therefore remain the standard of care,^7^^,^^8^ but they often incur substantially higher costs, owing to additional reagents, instrumentation, and technical processing.

On the other hand, digital histology (and histopathology) and the use of whole-slide images (WSIs) have recently shown promising accuracy in capturing nuanced patterns that human observers often miss; however, they are often lacking in interpretability, limiting their clinical adoption. As a result, there is a pressing need for the best of both worlds: computational histology methods that not only classify slides accurately but also reveal how predictions relate to recognizable, biologically grounded morphologic patterns, with stability across different staining, scanning, and institutional settings.

Recent deep learning methods, including vision foundation models, have achieved strong performance in WSI classification across diverse diagnostic and molecular tasks,^7^^,^^9^^,^^10^^,^^11^ but most models function as black boxes that offer limited interpretability on what histologic patterns are driving their predictions.^12^^,^^13^^,^^14^ Weakly supervised multiple instance learning (MIL) is a widely adopted framework for WSI analysis, and popular MIL models (e.g., ABMIL,^15^ CLAM,^16^ and TransMIL^17^) treat each slide as a large, heterogeneous collection of tiles with only slide-level labels. Widely used interpretability methods, such as attention heatmaps and Grad-CAM,^18^ indicate where a model attends but do not provide concept-level, human-understandable explanations of which histologic patterns drive its predictions. As a result, current approaches offer only coarse, qualitative cues and cannot identify the discrete, reproducible morphologic concepts present in WSIs. This limits biological insight, reduces reproducibility across sites, and weakens trust in model predictions, therefore limiting clinical deployment.

These interpretability limitations motivate a closer examination of how MIL models encode morphology internally to determine whether their representations can be reorganized into clinically meaningful and human-understandable concepts. Prior work has shown that deep neural networks naturally organize intermediate features into latent spaces that encode semantic or visual factors.^19^^,^^20^ In attention-based MIL (ABMIL) models, the tile-level embeddings produced before attention pooling define an intermediate *h*-space that captures the morphologic features learned by the model across tumor and stromal regions. Latent spaces such as the *h*-space can be reorganized into human-interpretable concepts,^21^ and concept discovery from neural embeddings has been shown to recover coherent visual structures.^22^^,^^23^^,^^24^^,^^25^^,^^26^ Together, these observations suggest that MIL backbones already encode rich morphologic structure but require an attention-aware organization strategy to make this structure explicit and biologically interpretable.

In this work, we show that attention mechanisms in MIL induce a latent morphologic structure that can be reorganized into discrete histologic concepts without tile-level annotations. We develop CLEAR-HPV (concept-level explainable attention-guided representation for HPV), a framework that restructures the attention-weighted (AW) MIL *h*-space to enable annotation-free concept discovery in HPV-related histopathology. Rather than modifying the classifier or explicitly optimizing for higher accuracy, CLEAR-HPV operates post hoc on the latent embeddings of a *trained* model (e.g., CLAM^16^), using attention weights to focus concept discovery on tiles the model already considers informative. The framework yields coherent keratinizing, basaloid, and stromal morphologic concepts that align with established HPV-associated patterns, along with two complementary interpretable outputs: spatial concept maps, which show where concepts appear across each slide, and compact concept-fraction representations, which summarize slide-level tissue composition in a quantitative, low-dimensional form.

**Figure 1:**
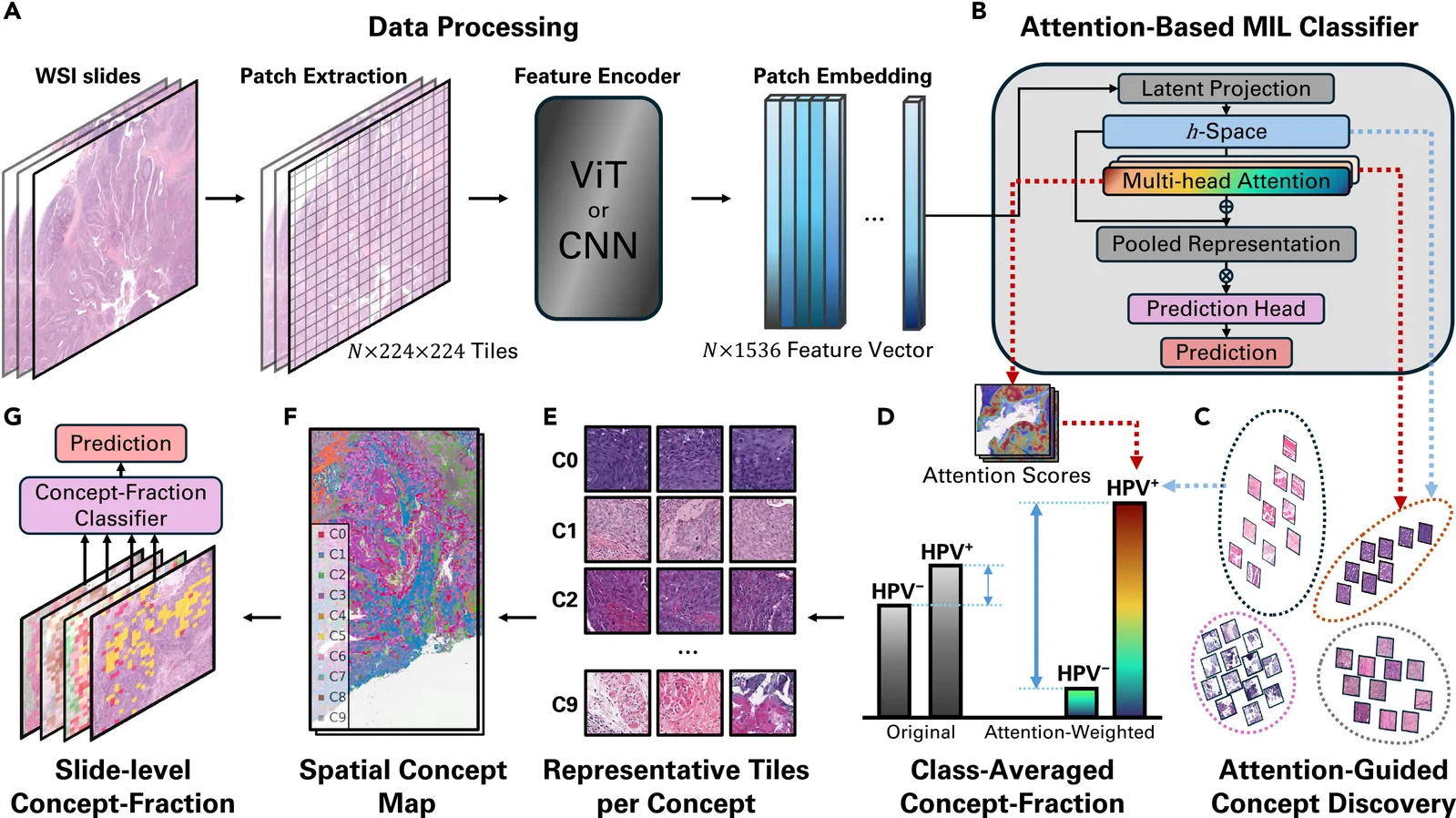
Overview of the CLEAR-HPV framework (A) Data processing pipeline: WSIs are decomposed into fixed-size tiles, encoded with a pretrained ViT or CNN, and converted into patch-level feature embeddings. (B) An attention-based MIL classifier projects embeddings into the *h*-space latent representation and uses multi-head attention to compute tile-level contributions, which are pooled into a single slide-level embedding for HPV prediction. (C) CLEAR-HPV performs annotation-free concept discovery on attention-weighted *h*-space embeddings to identify coherent morphologic concepts. (D) Using the discovered concepts, each slide is represented by a concept-fraction vector, which is then averaged across slides to obtain class-averaged concept-fraction vectors that summarize morphologic composition for HPV-positive and HPV-negative cohorts. (E) Representative tiles illustrate the characteristic morphology captured by each discovered concept. (F) Spatial concept maps visualize the distribution of concepts across the WSIs, revealing their spatial organization. (G) Slide-level concept-fraction vectors provide an interpretable representation that supports a concept-fraction classifier, which recovers MIL predictive performance while offering concept-level explanations. More details are available in the methods section.

Applied to three cohorts of data, The Cancer Genome Atlas (TCGA) head and neck squamous cell carcinoma (HNSCC),^27^ cervical squamous cell carcinoma and endocervical adenocarcinoma (CESC),^27^ and Clinical Proteomic Tumor Analysis Consortium (CPTAC)-HNSCC,^28^ CLEAR-HPV discovers stable concepts and consistent concept-fraction patterns that generalize across cohorts, indicating that the discovered morphology reflects consistent HPV-associated structure rather than dataset-specific artifacts. The resulting concept-fraction representations preserve the discriminative structure encoded in the original MIL embeddings, allowing downstream classifiers to recover comparable slide-level predictions while operating on an interpretable concept space—achieving the best of both worlds. Together, these findings demonstrate that the latent feature space of ABMIL already contains rich, biologically meaningful organization and that our attention-guided concept discovery can expose this structure without sacrificing predictive performance. Figure 1 shows an overview of our CLEAR-HPV, and implementation details are described in the methods section.

In summary, our primary contributions are as follows.

(1) We introduce CLEAR-HPV, the first general framework to automatically discover pathology-relevant morphologic concepts for HPV prediction without tile-level supervision (or annotation).
(2) We demonstrate that CLEAR-HPV leverages the AW latent space in a deep learning model to produce spatial concept maps and concept-fraction vectors, offering biologically grounded, concept-level interpretability for whole-slide histopathology.
(3) CLEAR-HPV preserves the predictive performance of the interpreted model while reducing its high-dimensional features (e.g., 1,536 dimensions) to only 10 interpretable concepts.
(4) We show that CLEAR-HPV is compatible with diverse ABMIL backbones and retains strong slide-level performance across diverse architectural designs, enabling robust and consistent concept discovery beyond a single model instantiation.
(5) We further show that these concept-level representations are stable across different cohorts (e.g., TCGA and CPTAC), preserve clinically relevant predictive signal under transfer, and reveal cross-cohort consistency of HPV-related morphology.

## Results

In this study, we evaluated CLEAR-HPV across three independent WSI cohorts (datasets) to examine whether biologically coherent and interpretable concepts can be discovered across diverse clinical and technical settings. The TCGA-HNSCC cohort included 102 patients and 106 diagnostic WSIs (38 HPV positive and 64 HPV negative). The TCGA-CESC cohort consisted of 146 patients and 154 WSIs, predominantly HPV positive (138 HPV positive and 8 HPV negative).^27^ The CPTAC-HNSCC dataset contributed 112 HPV-negative patients and 368 WSIs, serving as an external validation cohort collected under a different study protocol.^28^ These datasets differ substantially in HPV prevalence, staining and scanning protocols, and clinical outcomes, allowing us to assess whether CLEAR-HPV identifies stable morphologic concepts rather than cohort-specific artifacts.

CLEAR-HPV is designed as a post hoc explainability framework; its goal is *not* to improve predictive accuracy but to discover and interpret the morphologic concepts encoded in the model’s internal representations. Compared to the interpreted (explained) deep learning model that uses high-dimensional (e.g., 1,536 dimensions), uninterpretable embeddings, CLEAR-HPV discovers a compact, interpretable set of concepts (e.g., only 10 concepts). Therefore, results are considered very strong as long as comparable predictive performance (e.g., area under the curve [AUC], accuracy [ACC], and F1) can be achieved using CLEAR-HPV’s discovered concepts.

We use CLAM,^16^ a widely adopted ABMIL method, as the primary target backbone (base) model to explain. CLAM provides tile-level attention scores and a learned intermediate latent space (the *h*-space). Together, these form the foundation for concept discovery. We also provide results for three other backbone models and their variants to demonstrate the generality of CLEAR-HPV (Table 3).

A consistent 10-fold protocol was applied to each cohort, enabling systematic assessment of interpretability and robustness across heterogeneous datasets (Figure 1).

### Base model performance

Table 1 shows that, on TCGA-HNSCC, the CLAM ABMIL backbone achieved consistent slide-level performance (ACC = 0.77 ± 0.06, AUC = 0.86 ± 0.05), indicating that its learned representations capture generalizable, discriminative structure.

**Table 1:** Cross-cohort generalization of the baseline CLAM model

Dataset	ACC	AUC	Prec	Rec	F1
TCGA-HNSCC	0.765 ± 0.063	0.863 ± 0.051	0.788 ± 0.098	0.673 ± 0.145	0.696 ± 0.101
TCGA-CESC	0.593 ± 0.083	0.684 ± 0.154	0.977 ± 0.029	0.581 ± 0.176	0.716 ± 0.073
CPTAC-HNSCC^a^	0.704 ± 0.120	N/A	N/A	N/A	N/A

a AUC, precision, recall, and F1 are not applicable (N/A) because the CPTAC-HNSCC cohort contains only HPV-negative slides (single class).

We then evaluated the model in a fully zero-shot setting on external cohorts without any calibration or retraining. On TCGA-CESC, a cohort dominated by HPV-positive tumors, performance decreased as expected due to differences in tissue origin and histomorphologic context (AUC ≈ 0.68), but the model retained very high precision (Prec ≈ 0.98), indicating preservation of class-specific structure under domain shift. On CPTAC-HNSCC, where only HPV-negative cases are available for evaluation, ACC remained consistent (0.70 ± 0.12), demonstrating robustness to moderate staining and scanner variability. Together, these zero-shot evaluations show that the CLAM backbone maintains consistent decision behavior across cohorts and preserves transferable class-related structure in its latent representation, making it suitable for downstream concept-level analysis.

### Annotation-free concept discovery in the *h*-space

CLEAR-HPV is motivated by the observation that the latent *h*-space of ABMIL models contains a rich morphologic structure that can be made explicit via concept discovery. Here, the *h*-space consists of tile-level embeddings produced before attention pooling, illustrated as a blue box in Figure 1B. The attention weights learned by the backbone identify which regions contribute most to the final prediction, and attention pooling is typically used to pool these tile-level embeddings into a single embedding vector (i.e., “pooled representation” in Figure 1B), which is fed into a prediction head for classification. CLEAR-HPV constructs an attention-guided representation by weighting each embedding *h*_*i*_ by its attention score *α*_*i*_, thereby emphasizing diagnostically informative tiles while reducing background variation. More details are provided in the methods section.

#### Concept-discovery methods and baselines

To evaluate the discovered concepts, all concept-discovery methods were applied to TCGA-HNSCC training folds, which provide sufficient morphologic diversity for assessing cluster structure. Concept discovery was performed with *K* = 10 concepts. This choice is supported by consistent empirical evidence: Table S1 shows that predictive performance remains stable across *K* ∈ {5,10,15} with overlapping confidence intervals across all major metrics, indicating that *K* = 10 achieves comparable performance without sensitivity to the exact choice of *K*. Table S2 further demonstrates that concept geometry is highly stable across resolutions, with forward persistence and reverse fragmentation both exceeding 0.96, confirming that the discovered concepts are preserved under changes in *K*. The elbow analysis (Figure S1) provides additional support, showing diminishing returns in clustering compactness beyond *K* = 10. We evaluate two variants of CLEAR-HPV: concepts produced from raw-*h*-space embeddings, i.e., “CLEAR-HPV (raw-*h*),” and concepts derived from AW *h*-space embeddings, i.e., “CLEAR-HPV (AW-*h*).” These form the primary concept sets used throughout the analysis. For comparison, we evaluated several baseline methods that differ in where and how morphologic structure is extracted. Specifically, we considered the following (more details are provided in the methods section): (1) heatmap-based grouping, which reflects which tiles the model attends to, without defining discrete concept distributions; (2) encoder-feature clustering, which generates unconstrained concept distributions derived from encoder feature representations; and (3) a Dirichlet concept model, which represents concept membership probabilistically and allows tiles and slides to express varying degrees of concept mixing. Together, these baselines compare different input representations and concept construction approaches, highlighting how attention-structured *h*-space representations influence the coherence and interpretability of discovered morphologic concepts.

#### Quantitative comparison of concept-discovery methods

After defining the set of concept-discovery methods, we evaluate all approaches under a common framework to assess how well the resulting concepts summarize slide-level morphology and preserve diagnostically relevant signals. With *K* concepts in total, each slide is summarized by a *K*-dimensional concept-fraction vector that quantifies the proportion of tiles assigned to each concept. The concept-fraction vector is the core representation used throughout our analysis, providing a unified and interpretable summary of slide-level morphology derived from tile-level concepts. To assess whether these concepts capture diagnostically meaningful information, we introduce a concept-fraction classifier that maps concept-fraction vectors to HPV status, without introducing additional trainable parameters.

A detailed description is provided in the methods section. We use common classification metrics such as ACC, AUC, Prec, recall, and F1 to measure how well each representation preserves the predictive signal present in the original MIL embeddings. Note that our goal is *not* to improve ACC on the original MIL backbone (e.g., CLAM). Instead, we aim to measure how well the concept-based explanations can retain the predictive performance of the original MIL backbone for HPV status. Therefore, we consider these results strong when the CLEAR-HPV predictor achieves performance (e.g., ACC or AUC) comparable to the original MIL backbone.

Table 2 shows the performance of HPV classification for all concept-discovery methods using only the discovered concept-fraction vectors, evaluated with a simple rule-based concept-fraction classifier. Our CLEAR-HPV’s two variants, raw-*h* and AW-*h*, capture complementary structures. Raw-*h* preserves the intrinsic latent space learned by the MIL encoder and achieves the highest AUC, while AW-*h* produces more coherent and stable morphologic concepts by amplifying high-attention tiles. Notably, even using only the discovered concept-fraction vector (with only *K* = 10 dimensions), without access to the original tile embeddings (with 1,536 dimensions), both variants retain predictive performance comparable to the CLAM backbone, indicating that the concept-fraction representation preserves the discriminative signal of the original model despite substantial dimensionality reduction.

**Table 2:** Comparison of concept-discovery methods on TCGA-HNSCC

Method	ACC	AUC	Prec	Rec	F1
Heatmap	0.538 ± 0.110	0.674 ± 0.147	0.503 ± 0.137	0.606 ± 0.215	0.538 ± 0.114
Dirichlet concepts	0.646 ± 0.071	0.641 ± 0.134	0.475 ± 0.314	0.200 ± 0.208	0.266 ± 0.193
Encoder concepts	0.709 ± 0.126	0.847 ± 0.139	0.648 ± 0.162	0.835 ± 0.157	0.714 ± 0.109
CLEAR-HPV (AW-*h*)	0.784 ± 0.141	0.843 ± 0.117	0.797 ± 0.197	0.623 ± 0.284	0.715 ± 0.206
CLEAR-HPV (raw-*h*)	0.749 ± 0.119	0.889 ± 0.078	0.770 ± 0.181	0.633 ± 0.204	0.684 ± 0.146

The heatmap provides an attention-only reference and does not define discrete concepts. Encoder concepts derive clusters directly from encoder feature representations. Within the *h*-space, Dirichlet concepts serve as an alternative concept-discovery baseline applied to the unweighted latent space. In contrast, CLEAR-HPV performs concept discovery directly in the attention-weighted latent *h*-space. Metrics assess whether the resulting concept-fractions vectors retain the predictive signal present in the original MIL backbone. Rec, recall. Results on more metrics are provided in Table S3.

Baselines that do not operate in the *h*-space performed substantially worse. We find that heatmap-based grouping produced diffuse partitions with limited HPV separation, indicating that spatial saliency alone is insufficient for isolating meaningful morphologic patterns; the Dirichlet model emphasizes either global regularity or high flexibility, which can conflict with the localized, heterogeneous, and uneven distribution of HPV-associated morphologic patterns in histopathologic tissue, making these distributions difficult to fit reliably under weak slide-level supervision; encoder-based clustering achieved reasonable quantitative performance but showed weaker class-coherent organization than *h*-space-based concept discovery, both qualitatively and quantitatively. In particular, the added class-dominance analyses in Figures 3E and 3F show that encoder-space concepts exhibit substantially lower dominant-cluster purity and mass concentration than CLEAR-HPV concepts, indicating greater mixing of class-relevant morphology within clusters. Both metrics are defined in methods S1.

#### Recovery score: How well CLEAR-HPV retains predictive performance

**Figure 2:**
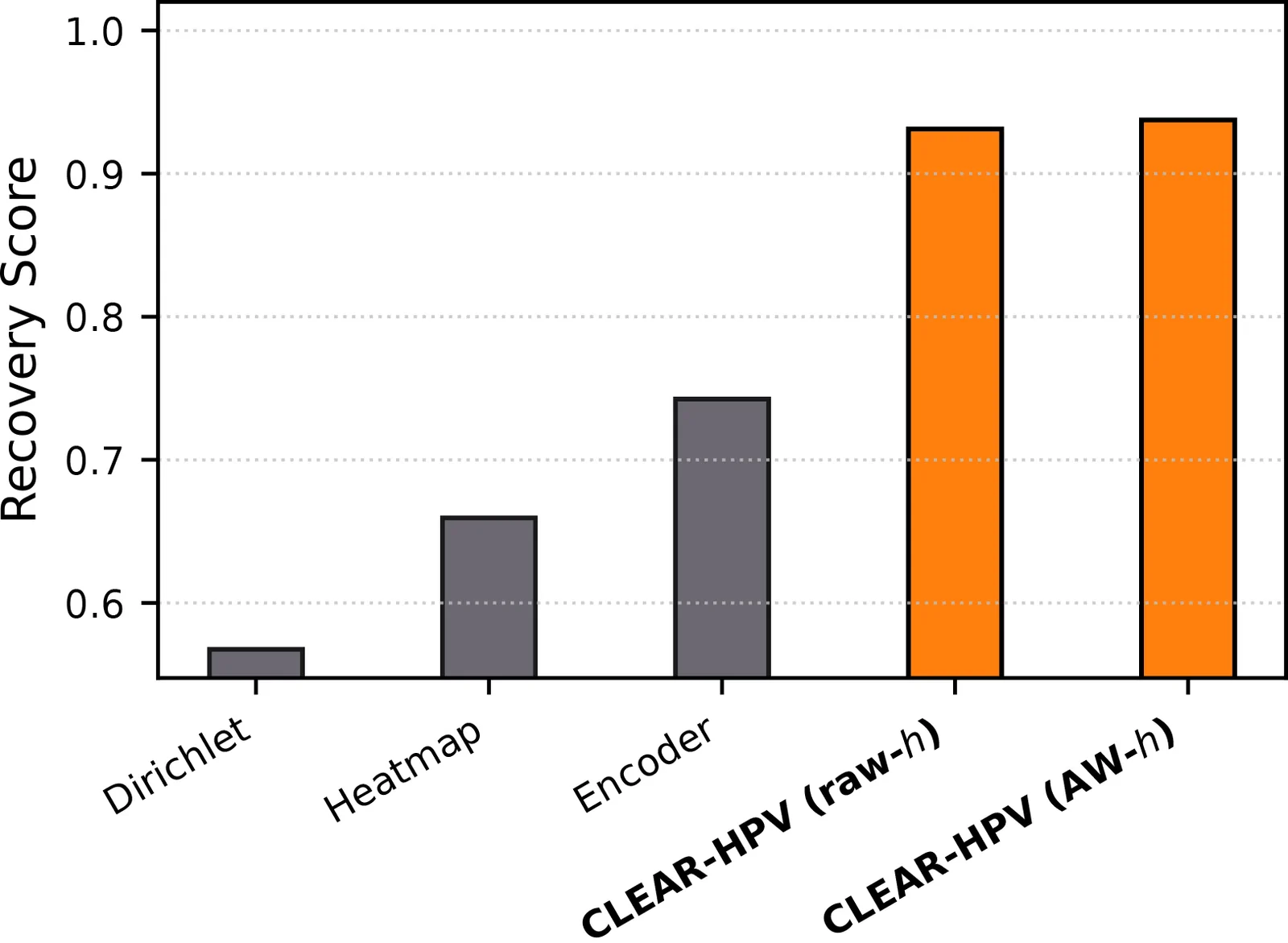
Recovery score relative to the interpreted MIL model (CLAM) across ACC, AUC, F1, precision, recall (i.e., sensitivity), and specificity For each method, the Euclidean distance *d* between its metric vector (i.e., concatenation of accuracy, AUC, etc.) and that of the interpreted model is computed and converted to a similarity score $s=\frac{1}{1+d}$. Higher scores indicate closer agreement with CLAM.

To further assess how well the discovered concepts retain the predictive performance of the backbone MIL model, we also compute a “recovery score” that quantifies how much predictive performance (e.g., ACC and AUC) can be recovered using only the discovered concepts from different methods (including our CLEAR-HPV). (Details on how to compute the recovery score are included in the methods section.) Figure 2 shows the results. CLEAR-HPV (AW-*h*) and CLEAR-HPV (raw-*h*) achieve the highest recovery scores, demonstrating that AW latent structure supports faithful, interpretable concept decompositions. Baselines such as heatmap-based grouping and Dirichlet clustering achieve much lower recovery scores, indicating a limited ability to preserve the original model’s predictive performance.

Together, these results show that concept discovery is most effective when performed directly in the attention-structured *h*-space and that CLEAR-HPV can provide high-quality explanations of an MIL backbone’s prediction by retaining its discriminative signal while providing compact, interpretable concept-based representations. These are strong results because they show that CLEAR-HPV (1) preserves the predictive performance of the interpreted model while reducing its high-dimensional features (e.g., 1,536 dimensions) to only 10 interpretable concepts and (2) improves class-coherent organization of discovered concepts, as quantified by our class-dominance metrics.

### Interpretability

While the preceding section evaluated how well different concept-discovery methods preserve predictive signal and performance using quantitative classification metrics, these metrics alone do not explain what morphologic patterns the models rely on. We therefore next examine the interpretability of the discovered concepts by analyzing how they are distributed across slides and how clinicians can use these concept-fraction vectors. This analysis shifts the focus from performance to biological meaning, asking whether the learned concepts correspond to coherent and clinically recognizable histopathologic patterns.

#### Class-averaged concept-fraction analysis

To facilitate interpretability (explainability), we analyze concept-fraction vectors at the cohort level by averaging slide-level concept-fraction vectors within each group (e.g., HPV-positive and HPV-negative patients). These class-averaged concept-fraction vectors provide a compact quantitative summary of morphologic composition that can be directly compared across HPV status, survival outcomes, and cohorts. For example, an average concept-fraction vector over HPV-positive patients provides insight into which concepts are most relevant to positive predictions, and similarly for HPV-negative patients. A larger difference between these two vectors indicates that the discovered CLEAR-HPV concepts better distinguish HPV-positive from HPV-negative cases.

**Figure 3:**
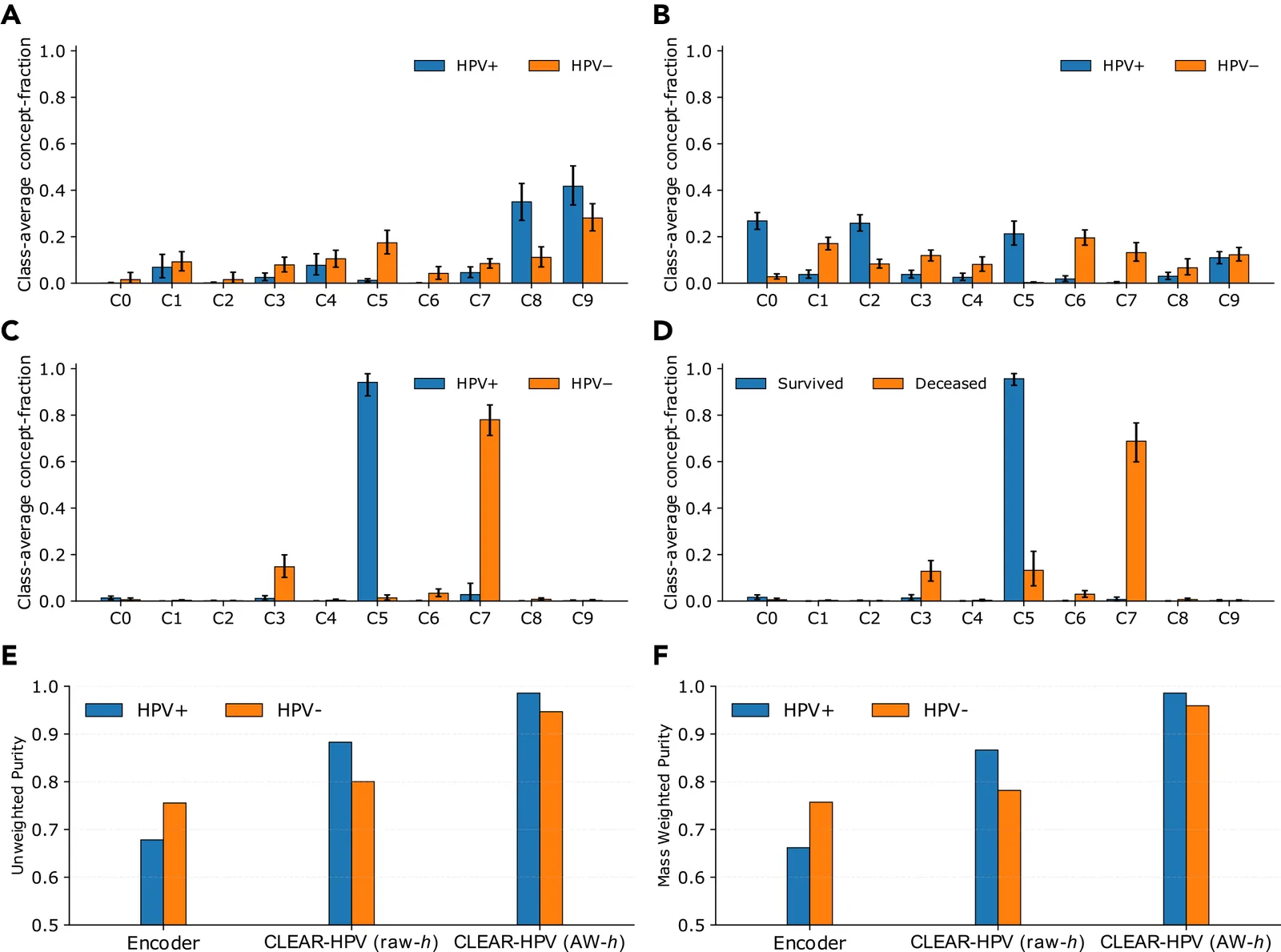
Class-averaged concept-fraction vectors across concept-discovery settings on TCGA-HNSCC Concept-fraction vectors are computed per slide as the fraction of tiles assigned to each discovered concept, optionally weighted by MIL attention. These slide-level vectors are then averaged within each group to obtain class-averaged profiles that summarize cohort-level morphologic composition and highlight differences in concept prevalence across clinical groups. (A)–(C) show group-averaged profiles for HPV-positive (blue) and HPV-negative (orange) cases, while (D) shows the corresponding averages for surviving (blue) and deceased (orange) cases. Error bars denote 95% bootstrap confidence intervals of the class mean computed across slides within each group. (A) Class-averaged concept-fraction vectors derived from encoder-feature clustering (non-*h*-space baseline). (B) Class-averaged concept-fraction vectors derived from CLEAR-HPV concepts in the MIL *h*-space using unweighted fractions, where all tiles contribute equally. (C) Class-averaged concept-fraction vectors derived from CLEAR-HPV concepts in the MIL *h*-space using attention-weighted fractions, where each tile contributes proportionally to its MIL attention score, yielding clearer separation between HPV-positive and HPV-negative cases. (D) Using the same CLEAR-HPV *h*-space concepts and attention-weighted fractions as in (C), class-averaged concept-fraction vectors are shown for surviving versus deceased cases. (E) Unweighted purity (cluster-averaged class dominance) across methods, computed as the mean class-dominance ratio over clusters dominated by each class. (F) Mass weighted purity (dominant-cluster class-mass concentration) across methods, computed as the fraction of class-specific mass within clusters dominated by each class; higher values indicate stronger class coherence. Detailed definitions are provided in methods S1.

**Figure 4:**
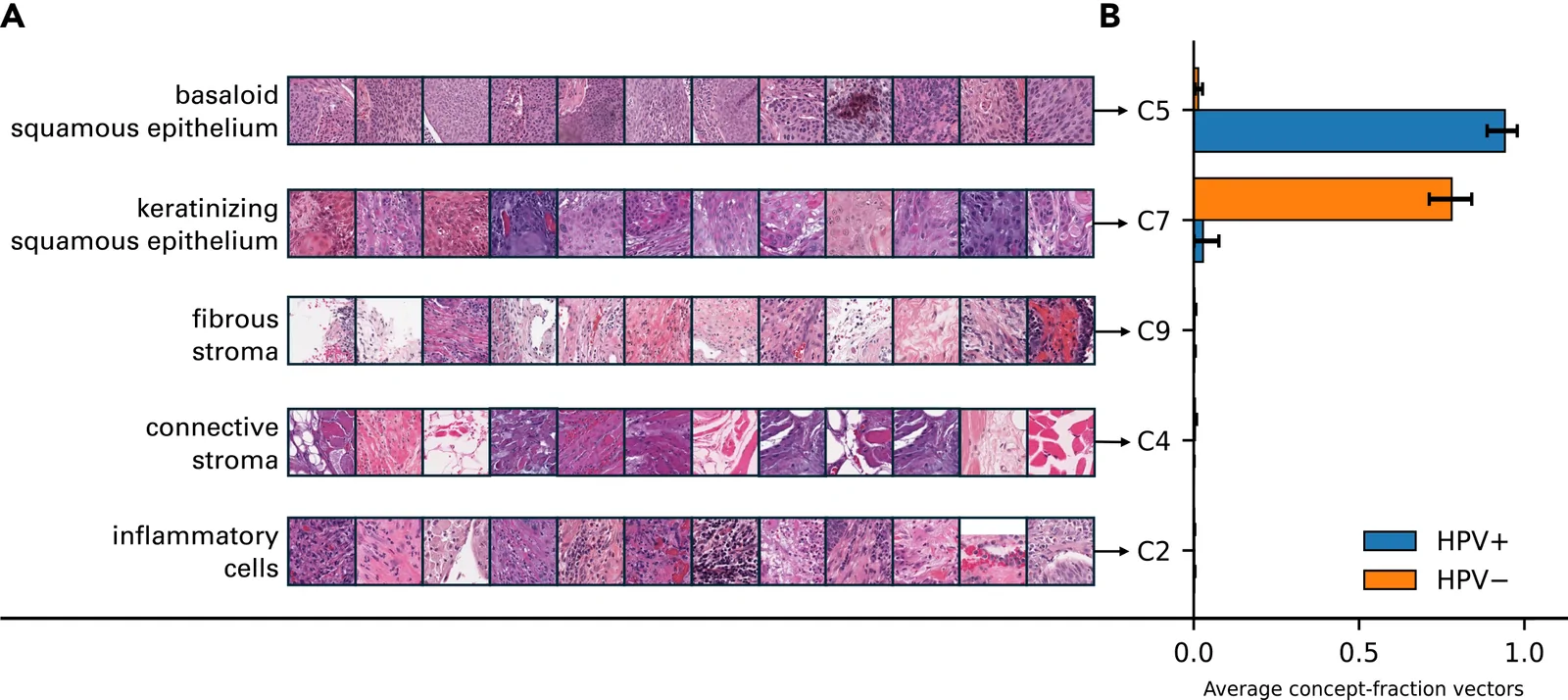
Top tiles for key concepts discovered by CLEAR-HPV and the corresponding slide-level distributions in the dataset TCGA-HNSCC (A) Top (representative) tiles for five CLEAR-HPV concepts chosen for their consistent appearance and clear morphologic identity: C5 (basaloid squamous epithelium), C7 (keratinizing squamous epithelium), C9 (fibrous stroma), C4 (connective stroma), and C2 (inflammatory cells). (B) Average concept-fraction vectors for HPV-positive (blue) and HPV-negative (orange) slides, with 95% bootstrap confidence intervals. The basaloid concept C5 is more prevalent in HPV-positive cases, while the keratinizing concept C7 is more prevalent in HPV-negative cases.

Figure 3 shows the class-averaged concept-fraction vectors for HPV-positive and HPV-negative cases with *K* = 10 concepts (C0–C9). Encoder-space concepts (Figure 3A) show little separation between classes. For CLEAR-HPV concepts discovered in the MIL *h*-space, unweighted concept fractions (Figure 3B; each tile contributes equally to its assigned concept) yield only modest class separation. In contrast, AW concept fractions (Figure 3C; each tile’s contribution to the slide-level fraction is weighted by its MIL attention score) produce a clearer dichotomy: HPV-positive slides show higher fractions of the basaloid/non-keratinizing concept (concept 5 [C5]), whereas HPV-negative slides show higher fractions of the keratinizing concept (C7). Representative tiles supporting these morphologic interpretations are shown in Figure 4, consistent with established histopathologic differences. Using the same AW fraction computation, Figure 3D shows a clear survival-associated discrepancy in concept composition. Survivors are enriched for C5, whereas deceased cases are enriched for C7. This pattern is consistent with the established prognostic advantage of HPV-driven tumors, even though the MIL backbone was trained only for HPV prediction.

To quantify the class-coherent structure of discovered concepts, we introduce two metrics based on class-averaged concept-fraction vectors: unweighted purity, which measures the mean purity of class-dominant clusters, and mass-weighted purity, which emphasizes whether class-specific signal is concentrated in high-mass clusters. Detailed definitions are provided in methods S1. Both metrics consistently show that AW CLEAR-HPV produces higher class dominance and mass concentration than encoder-space concepts, indicating improved class-coherent organization of morphology.

#### Concept identity and representative morphology

Figure 4 shows representative tiles for key concepts discovered by CLEAR-HPV (Figure 4A) and the corresponding slide-level distributions in TCGA-HNSCC (Figure 4B). In Figure 4A, clinician review confirmed that these concepts correspond to inflammatory infiltrates (C2), benign stroma (C4), HPV-associated basaloid carcinoma (C5), HPV-negative keratinizing carcinoma (C7), and fibrous stroma (C9). These concepts appear consistently across datasets and form the basis for interpretable (explainable) slide-level composition profiles. In Figure 4B, we can see that the discovered concept C5 and concept C7 are highly relevant to HPV-positive and HPV-negative cases, respectively.

#### Slide-level visualization of concepts discovered by CLEAR-HPV

**Figure 5:**
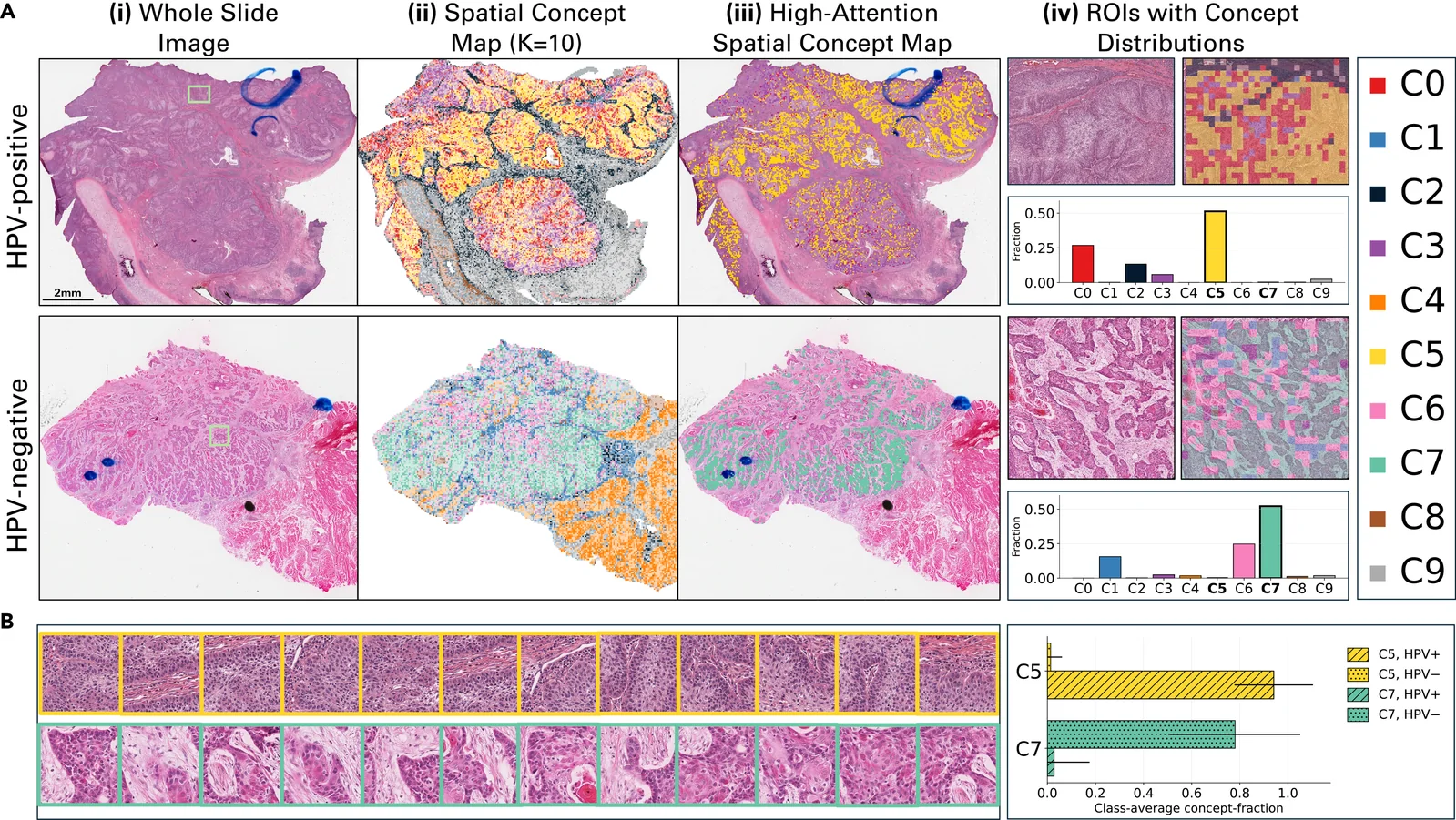
Visualization of attention-weighted concept discovery using CLEAR-HPV (A) For representative HPV-positive and HPV-negative WSIs from TCGA-HNSCC, we show, in four columns, (i) the original H&E WSI, (ii) the *h*-space spatial concept map, (iii) the high-attention spatial concept map, and (iv) regions of interest (ROIs) with their corresponding concept-fraction distributions produced by our CLEAR-HPV. (B) Representative tiles for two CLEAR-HPV concepts (C5 and C7), along with the average concept-fraction vectors for HPV-positive and HPV-negative slides of the entire dataset. Error bars denote 95% bootstrap confidence intervals of the class mean computed across slides within each group. We use different colors to represent different concepts consistently across all figures. Blue markings visible in the WSIs in (A) are pre-existing annotation artifacts on physical slides by clinicians and can be omitted from interpretation.

Figure 5 visualizes concepts within two representative HPV-positive and HPV-negative slides. High-attention maps (column 3 of Figure 5A) are obtained by ranking tile-level attention scores and retaining only top-scoring tiles, capturing regions most emphasized by the MIL backbone. Within these areas, HPV-positive slides consistently show the “basaloid” concept C5 (in yellow), while HPV-negative slides show the “keratinizing” concept C7. Background concepts such as fibrous or benign stromal tissue (e.g., C4 and C9), as indicated by their representative tiles in Figure 4, also appear consistently across slides and provide visual context for surrounding tumor regions. These representative tiles are selected by their distance from the concept center (see details in the methods section).

To quantify concept composition in highlighted, key areas of a slide, we define regions of interest (ROIs) as the contiguous high-attention regions outlined by the green box in Figure 5A(i), with a zoomed-in view shown in Figure 5A(iv). The concept-fraction distributions in the ROIs, shown in the green box in Figures 5A(i) and in Figure 5A(iv), match the dataset-level average concept-fraction vectors in Figure 5B (right): C5 is enriched in HPV-positive cases and C7 is enriched in HPV-negative cases. Together, these results show that (1) our CLEAR-HPV can provide slide-level concept explanations for HPV prediction while also highlighting important regions in the slide and (2) our CLEAR-HPV produces consistent concepts at the slide and dataset levels; slide-level average concept-fraction vectors within high-attention regions align with their dataset-level (global) average concept-fraction vectors across the cohort.

### Comparison of concept discovery across MIL backbones

Our CLEAR-HPV framework is compatible with arbitrary ABMIL neural network architectures. In addition to CLAM, we evaluated three MIL backbones, including two widely used MIL backbones, i.e., ABMIL^15^ and transformer-based MIL (TransMIL),^17^ as well as a multi-head attention-based MIL (MHMIL) model, to investigate how backbone choices influence the structure of the latent *h*-space and the resulting CLEAR-HPV concepts.

ABMIL employs a gated attention mechanism to learn instance-level importance weights and aggregates tile embeddings via a weighted sum, providing a simple and effective attention-based pooling strategy. TransMIL extends the MIL paradigm by modeling global contextual relationships among instances using transformer layers, enabling long-range dependency modeling across tiles within a WSI. MHMIL is inspired by CLAM; it replaces the single attention head with multiple parallel heads, allowing different heads to capture complementary tissue patterns while preserving the underlying MIL aggregation structure. By applying CLEAR-HPV to ABMIL, TransMIL, and MHMIL, we assess its robustness for concept discovery across distinct architectural inductive biases.

Using the same evaluation procedure as in the CLAM-based experiments, each slide is represented by a concept-fraction vector and evaluated with a concept-fraction classifier across all backbones. As shown in Table 3, CLEAR-HPV consistently preserves slide-level predictive performance across multiple ABMIL architectures, even though it replaces the original high-dimensional latent representations (i.e., 1,536 dimensions), which are *not* interpretable at the concept level, with substantially more compact (i.e., 10 dimensions) and interpretable concept representations. These are therefore strong results. Across ABMIL, TransMIL, and MHMIL, CLEAR-HPV achieves AUC (≈0.84–0.88) and ACC (≈0.76–0.81) that closely match those of the corresponding original backbone models (AUC ≈ 0.88–0.90, ACC ≈ 0.78–0.82). Importantly, we observe the following.

• Performance retention is consistent across architectures with distinct attention mechanisms and modeling complexity, indicating that the discovered concepts capture backbone-agnostic, diagnostically relevant morphology rather than architecture-specific artifacts.
• High performance is achieved when compressing the 1,536-dimensional representation from the MIL backbone to only 10 interpretable concept dimensions, demonstrating that CLEAR-HPV retains discriminative signal in a substantially more compact and interpretable representation, without reliance on high-dimensional, uninterpretable embeddings.

**Table 3:** Original backbone performance vs. CLEAR-HPV concept discovery across MIL backbones (on TCGA-HNSCC)

Backbone	Method	Feat. dim.	Interpret.	ACC	AUC	Prec	Rec	F1
**Original backbone performance (no concept discovery)**								

ABMIL	original	1,536	✗	0.818 ± 0.051	0.879 ± 0.062	0.867 ± 0.098	0.725 ± 0.104	0.765 ± 0.068
TransMIL	original	1,536	✗	0.784 ± 0.057	0.899 ± 0.052	0.800 ± 0.098	0.698 ± 0.085	0.734 ± 0.072
MHMIL	original	1,536	✗	0.791 ± 0.079	0.879 ± 0.068	0.825 ± 0.134	0.685 ± 0.160	0.723 ± 0.116

**CLEAR-HPV concept discovery**								

ABMIL	AW-*h*	10	✓	0.799 ± 0.087	0.859 ± 0.081	0.769 ± 0.115	0.767 ± 0.222	0.751 ± 0.133
ABMIL	raw-*h*	10	✓	0.810 ± 0.068	0.868 ± 0.086	0.811 ± 0.145	0.763 ± 0.169	0.769 ± 0.102
TransMIL	AW-*h*	10	✓	0.765 ± 0.077	0.863 ± 0.089	0.738 ± 0.071	0.703 ± 0.179	0.713 ± 0.111
TransMIL	raw-*h*	10	✓	0.784 ± 0.089	0.841 ± 0.084	0.803 ± 0.149	0.678 ± 0.147	0.727 ± 0.118
MHMIL	AW-*h*	10	✓	0.782 ± 0.145	0.859 ± 0.144	0.813 ± 0.229	0.669 ± 0.242	0.716 ± 0.204
MHMIL	raw-*h*	10	✓	0.794 ± 0.100	0.879 ± 0.129	0.859 ± 0.140	0.669 ± 0.273	0.713 ± 0.178

For each attention-based MIL backbone, we report the original backbone performance and the corresponding results obtained by applying CLEAR-HPV for concept discovery on the latent *h*-space. “Feat. dim.” denotes the dimensionality of the slide-level representation used for classification, and “Interpret.” indicates whether the representation yields explicit, human-interpretable morphologic concepts. Across all backbones, CLEAR-HPV achieves strong performance comparable to the original models. For MHMIL, performance metrics for individual attention heads and alternative head-aggregation strategies are provided in Table S4.

#### Encoder dependence

In addition to backbone robustness, we evaluated whether CLEAR-HPV depends on the choice of feature encoder by replacing the UNI pathology foundation model encoder (UNI)^29^ with a conventional ResNet50. CLEAR-HPV maintains comparable performance with this change and continues to produce coherent concept representations, indicating that the proposed framework is not tied to a specific foundation model encoder. Detailed results are provided in Table S5.

### Cross-cohort generalization and robustness

CLEAR-HPV concepts transfer reliably across datasets that differ in anatomic sites, staining profiles, and class distributions. All cross-cohort experiments were conducted in a zero-shot setting: concept clusters, attention weights, and the concept-fraction classifier were trained exclusively on TCGA-HNSCC and were not adapted, recalibrated, or tuned using any slides from TCGA-CESC or CPTAC-HNSCC. This design ensures that evaluations on the external cohorts measure genuine generalization rather than domain-specific adjustment. These results should be interpreted in terms of signal preservation and interpretability rather than improvement in predictive performance.

#### Transferring to the external cohort TCGA-CESC

**Figure 6:**
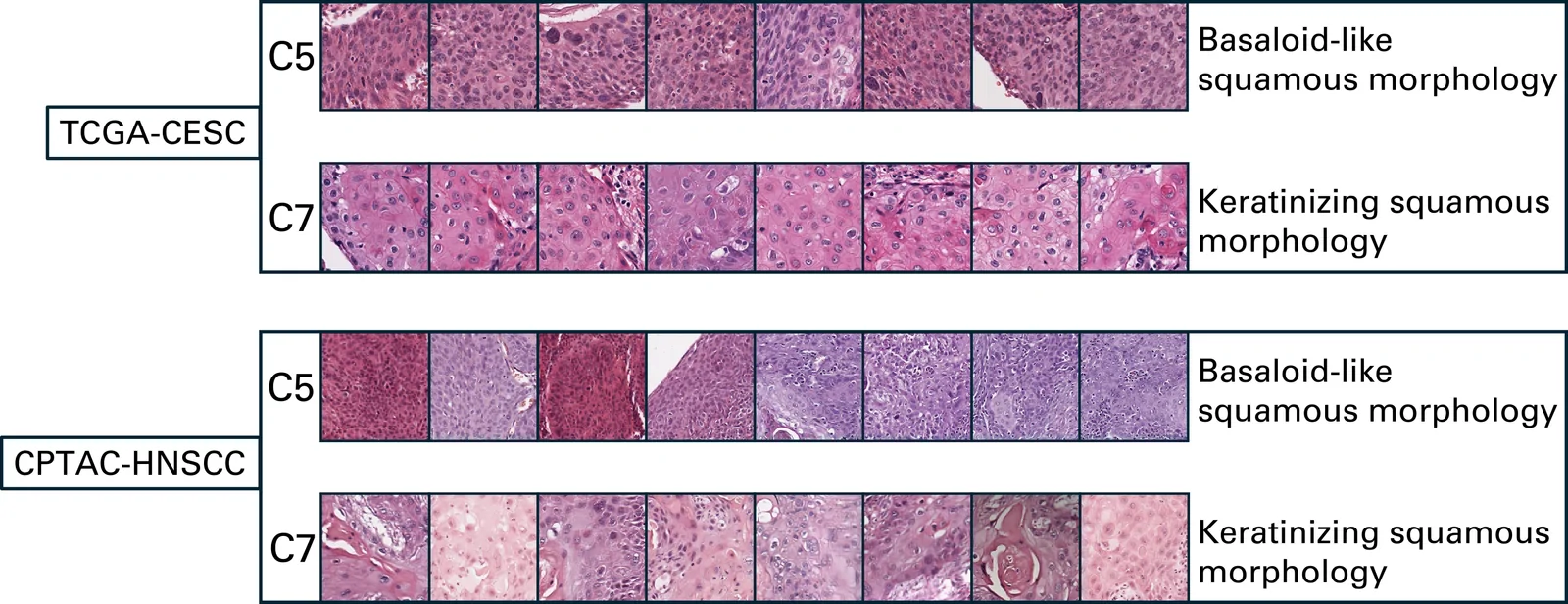
Cross-cohort consistency of HPV-related concepts among the top-8 tiles We show representative high-attention tiles for the HPV-positive-related basaloid concept C5 and the keratinizing concept C7 from two external cohorts, TCGA-CESC (top) and CPTAC-HNSCC (bottom). Across both datasets, C5 consistently reflects basaloid morphology characteristic of HPV-positive tumors, whereas C7 reflects keratinizing morphology typical of HPV-negative tumors. The consistency of these signatures across independent cohorts demonstrates that CLEAR-HPV identifies stable, biologically meaningful concepts that generalize beyond the training dataset.

Table 4 shows the results when models trained on TCGA-HNSCC were applied to the external cohort TCGA-CESC. Both CLEAR-HPV variants, AW-*h* and raw-*h*, retain substantial predictive signal under this substantial biological shift in tissue site. Note that TCGA-CESC differs substantially from TCGA-HNSCC in both anatomy and cohort composition: it is dominated by HPV-positive tumors and exhibits histomorphologic patterns distinct from HNSCC.^30^ Under this extreme imbalance, the CLAM baseline achieved only modest performance (ACC ≈ 0.59, AUC ≈ 0.68). Due to the strong class imbalance in TCGA-CESC, threshold-based metrics such as F1 and recall should be interpreted with caution, as they can be influenced by majority-class bias. Interestingly, both CLEAR-HPV variants, with their discovered interpretable concepts and concept-fraction vectors, maintain comparable AUC while achieving higher ACC (ACC ≈ 0.75) and, more importantly, preserve CLAM’s extremely high precision (Prec ≈ 0.96); they also achieved higher sensitivity and F1 than the CLAM base model. The AW variant achieved the highest F1 (≈0.86) and sensitivity (≈0.78), while the raw-*h*-space variant obtained the highest AUC (≈0.67) among CLEAR-HPV variants. However, AUC indicates a modest degradation in ranking performance under domain shift, consistent with our interpretation that CLEAR-HPV preserves interpretable structure rather than improving global discrimination. These results show that CLEAR-HPV captures HPV-related morphologic signals that remain predictive while maintaining interpretability under major shifts in tissue architecture. Together with the cross-cohort concept consistency shown in Figure 6, these results indicate that CLEAR-HPV preserves transferable morphologic structure even when ranking performance degrades modestly.

**Table 4:** Cross-cohort generalization from TCGA-HNSCC to TCGA-CESC

Method	ACC	AUC	Prec	Rec	F1
CLAM	0.593 ± 0.083	0.684 ± 0.154	0.977 ± 0.029	0.581 ± 0.176	0.716 ± 0.073
CLEAR-HPV (AW-*h*)	0.756 ± 0.069	0.650 ± 0.067	0.958 ± 0.013	0.777 ± 0.083	0.855 ± 0.049
CLEAR-HPV (raw-*h*)	0.748 ± 0.056	0.671 ± 0.061	0.960 ± 0.012	0.765 ± 0.066	0.850 ± 0.041

Under severe domain shift and strong HPV-positive class imbalance, CLEAR-HPV retains a substantial predictive signal within an interpretable concept space, although AUC indicates modest degradation in ranking performance relative to CLAM. Raw-*h* achieves the highest AUC among the CLEAR-HPV variants, while AW-*h* provides the highest F1 and recall. Results for additional metrics are provided in Table S6.

#### Transferring to the external cohort CPTAC-HNSCC

Similarly, Table 5 shows the results when models trained on TCGA-HNSCC were applied to the external cohort CPTAC-HNSCC, demonstrating CLEAR-HPV’s robustness to technical and institutional variability. Because this cohort contains only HPV-negative tumors, ACC is the only relevant metric. Both CLEAR-HPV variants outperformed CLAM (ACC ≈ 0.82–0.90), despite pronounced differences in staining, slide preparation, and scanner characteristics^28^ between the two cohorts. This improvement indicates that our CLEAR-HPV’s concept-fraction representations (vectors) remain stable under technical domain shift.

**Table 5:** External validation on CPTAC-HNSCC

Metric	CLAM	CLEAR-HPV (AW-*h*)	CLEAR-HPV (raw-*h*)
ACC	0.704 ± 0.120	0.824 ± 0.146	0.896 ± 0.094

Only accuracy is reported because the cohort contains only HPV-negative cases. CLEAR-HPV preserves the predictive information of the CLAM base model when transferred to an external cohort.

#### Morphologic consistency of concepts across cohorts

Figure 6 illustrates that the HPV-associated “basaloid” concept (C5) and the “keratinizing” concept (C7) retain their characteristic morphology in high-attention regions across both external cohorts, TCGA-CESC and CPTAC-HNSCC. The same concepts, basaloid tiles for C5 and keratinizing squamous morphology for C7, appear consistently, even in these external cohorts not seen during training. These cross-cohort results demonstrate that CLEAR-HPV captures stable, biologically meaningful morphologic structures that generalize across different squamous tumor types and institutional settings.

### Survival prediction based on CLEAR-HPV concepts

HPV status is a well-established prognostic marker in squamous cell carcinoma, with HPV-positive tumors exhibiting significantly better survival outcomes.^31^ We therefore assessed whether CLEAR-HPV concepts retain this prognostic signal by evaluating survival prediction in TCGA-HNSCC using only the concept-fraction vectors derived using CLEAR-HPV applied to an HPV-trained backbone. No retraining or survival-specific adaptation was performed; therefore, the survival model receives solely the morphologic composition learned from HPV supervision.

As shown in Table 6, the AW concept vectors produced by CLEAR-HPV achieve competitive, though slightly lower, predictive performance compared to the base model CLAM. While the explained model, CLAM, achieves the highest AUC (≈0.840), both CLEAR-HPV variants (which explain CLAM’s predictions) retain broadly comparable discriminative ability despite operating in a substantially lower-dimensional space. Note that these are already strong results because the CLEAR-HPV’s concept-fraction vectors have only 10 dimensions; they are both compact and interpretable. In contrast, the original CLAM model uses embeddings with 1,536 dimensions, which are not interpretable.

**Table 6:** Survival prediction on TCGA-HNSCC

Method	ACC	AUC	Prec	Rec	F1
CLAM	0.734 ± 0.086	0.840 ± 0.081	0.668 ± 0.160	0.673 ± 0.188	0.628 ± 0.143
CLEAR-HPV (AW-*h*)	0.715 ± 0.121	0.785 ± 0.134	0.665 ± 0.209	0.558 ± 0.147	0.594 ± 0.168
CLEAR-HPV (raw-*h*)	0.700 ± 0.139	0.766 ± 0.114	0.675 ± 0.229	0.530 ± 0.198	0.563 ± 0.192

Survival prediction using CLEAR-HPV concept representations (i.e., concept-fraction vectors) is compared with the base model CLAM. Results show that CLEAR-HPV’s concept-fraction vectors preserve the prognostic information contained in the original MIL embeddings of CLAM. Results for more metrics are provided in Table S7.

These results indicate that CLEAR-HPV concepts capture broader morphologic structure that remains informative for patient outcome, even though the concepts were originally learned for HPV prediction. The concept-fraction vectors provide a compact and interpretable summary of tumor and stromal composition, allowing links between morphologic patterns and adverse prognosis to be examined. Together, these findings show that reorganizing the MIL latent space into CLEAR-HPV concepts preserves survival-relevant information, even when supervision is based entirely on HPV labels.

## Discussion

This study demonstrates that the latent space learned by ABMIL models contains biologically structured information that becomes interpretable when it is reorganized through our proposed CLEAR-HPV framework. The latent *h*-space encodes tile-level morphology before slide-level aggregation. Weighting these embeddings by learned attention scores highlights the regions most relevant to the prediction and enables CLEAR-HPV to recover consistent and generalizable morphologic concepts without tile-level supervision or human annotation. CLEAR-HPV is not designed to improve classification accuracy but to provide a structured and interpretable view of the latent space learned by the model.

Our findings further demonstrate that CLEAR-HPV is not tied to a specific attention mechanism or MIL architecture but instead can be applied consistently across diverse attention-based backbones. Despite differences in model architecture and attention formulation, CLEAR-HPV relies on the attention learned for the prediction task to organize the latent *h*-space, resulting in stable and robust morphologic concept discovery. This architectural robustness indicates that CLEAR-HPV functions as a general concept-discovery framework that can be integrated with a wide range of ABMIL models without requiring model-specific modification.

CLEAR-HPV concepts demonstrate cross-cohort consistency across TCGA-HNSCC, TCGA-CESC, and CPTAC-HNSCC in a strict zero-shot transfer setting. Despite substantial variation in staining, scanner characteristics, and cohort composition,^32^^,^^33^ the same basaloid and keratinizing concepts appeared consistently across cohorts (datasets). This reproducibility and stability indicate that CLEAR-HPV captures transferable morphologic signals associated with HPV status rather than dataset-specific artifacts, even under substantial domain shift, although some degradation in ranking performance is observed in certain transfer settings. Importantly, these results reinforce that CLEAR-HPV is designed to preserve predictive signals in an interpretable representation rather than to optimize classification performance; thus, minor changes in standard metrics should be interpreted in the context of this trade-off.

The concept-fraction vectors produced by CLEAR-HPV also retained prognostic information (important for survival analysis) in TCGA-HNSCC, even though the concepts were learned solely from HPV supervision without any concept-level annotation or supervision. This shows that reorganizing the MIL latent space can preserve broader tumor biology and that interpretable concept profiles can serve as compact descriptors for downstream clinical tasks.

While recent progress in pathology foundation models (e.g., contrastive histology encoders,^34^ TCv2,^35^ UNI,^29^ CONCH,^36^ and GigaPath^37^) provides powerful feature extractors trained at scale, our results highlight that expressive embeddings alone do not guarantee interpretability (or explainability). Direct clustering of foundation-model features produced reasonable quantitative performance but did not reveal coherent morphologic structure. The AW *h*-space, by contrast, combines the representational richness of these encoders with task-specific focus, enabling concept discovery that aligns with the regions most relevant to the prediction. This intermediate representation offers a practical pathway toward interpretable foundation-model pipelines.

While CLEAR-HPV organizes latent representations into structured and quantitatively coherent concepts, assigning semantic labels to these concepts currently requires expert interpretation. Future work may explore the integration of large language models (LLMs) or multimodal vision-language models to assist with automatic concept description or naming, for example, by summarizing representative tiles associated with each concept. Such approaches could improve scalability and usability, but any automatically generated annotations would still require expert validation for clinical use.

In summary, CLEAR-HPV provides an interpretable link between slide-level predictions and human-understandable histology by restructuring the attention-mediated latent space into discrete morphologic concepts. These concepts are biologically coherent, reproducible across cohorts, and informative for classification and prognosis. More broadly, our findings show that the attention-structured latent space can support concept-level interpretability in whole-slide imaging across diverse MIL backbone architectures, offering a general framework for interpretable MIL. We expect that similar strategies will extend to additional molecular and prognostic tasks and will contribute to interpretable representation learning in computational pathology.

### Limitations of the study

This study has several limitations. First, the quality of the recovered concepts is influenced by the choice of MIL backbones; models with diffuse and weaker attention may offer slightly weaker structures for unsupervised separation of different concepts. Second, clustering is performed in a latent space without tile-level labels; therefore, certain distinctions may remain subtle and benefit from expert interpretation. In particular, clinically actionable interpretation of the discovered concepts requires expert pathology review. Third, our pipeline relies on pretrained encoders and fixed attention maps, following the standard histopathology MIL paradigm (e.g., CLAM), where tile encoders are not jointly optimized during slide-level training. A unified end-to-end optimization of the encoder, attention mechanism, and concept structure may yield more refined and semantically consistent concepts and remains an important direction for future work. Future work will incorporate spatial context, richer supervisory signals, and multimodal information to expand the biological insight provided by the CLEAR-HPV framework.

## Methods

Figure 1 shows the overview of our CLEAR-HPV framework. WSIs are first decomposed into fixed-size tiles, encoded with a pretrained vision transformer (ViT) or convolutional neural network (CNN), and converted into patch-level feature embeddings (Figure 1A). An ABMIL classifier then projects embeddings into an *h*-space latent representation and uses multi-head attention to compute tile-level contributions, according to which tile-level embeddings are then pooled into a single slide-level embedding for HPV prediction (Figure 1B).

Given an arbitrary ABMIL backbone model above, our CLEAR-HPV then performs annotation-free concept discovery on AW *h*-space embeddings to identify coherent morphologic concepts (Figure 1C). Using the discovered concepts, CLEAR-HPV can do the following.

• Compute the class-averaged concept-fractions to quantify the relative abundance of each concept for HPV-positive and HPV-negative slides within a given cohort; using attention weights can produce concepts that better distinguish between HPV-positive and HPV-negative cases (Figure 1D).
• Generate representative tiles that illustrate the characteristic morphology captured by each discovered concept (Figure 1E).
• Generate spatial concept maps to visualize the distribution of concepts across the WSIs, revealing their spatial organization (Figure 1F).
• Obtain slide-level concept-fraction vectors to provide an interpretable representation that supports a concept-fraction classifier, which recovers MIL predictive performance while offering concept-level explanations (Figure 1G).

Below, we provide details on each aforementioned module, evaluation, and implementation.

### Cohort curation and pathology criteria

TCGA-HNSCC WSIs were curated by removing slides with poor staining, marked artifacts, or inadequate focus, using a combination of metadata filtering and manual quality inspection. For TCGA-CESC, we followed the pathology quality-control criteria described in TCGA^30^ and retained only diagnostic slides with adequate tumor content and acceptable image quality. CPTAC-HNSCC slides were included as provided, as this cohort had already undergone standardized acquisition and quality review through the CPTAC pipeline.

### Data and preprocessing

We used diagnostic WSIs from TCGA-HNSCC, TCGA-CESC, and CPTAC-HNSCC. Tissue regions were identified using standard color-based masking, and each WSI was tiled into non-overlapping patches (256 × 256) at the full-resolution layer (level 0), as shown in Figure 1A. For each tile, we extracted a fixed embedding $x_{i}\in {\mathbb{R}}^{D_{\text{in}}}$ using the pretrained UNI encoder^29^ (*D*_in_ = 1,536) without any color normalization. Each slide with *N* tiles is represented as an *N* × *D*_in_ matrix of tile features. We have run experiments with color normalization as well, but it did not make a difference, probably because the UNI encoder is already robust to different color schemes. Therefore, we chose to run all experiments without any color normalization.

### MIL backbones and the *h*-space

#### Different MIL backbones

We evaluated four ABMIL architectures: CLAM, ABMIL, TransMIL, and MHMIL. All models share a unified projection layer that maps encoder features into a common latent *h*-space with the same dimensionality, enabling direct and controlled comparison of the resulting representations across backbones (see Figure 1B for a typical example of an ABMIL model).

#### Attention-structured *h*-space

As shown in Figure 1B, we define the *h*-space (the blue box) as the intermediate embedding space produced by the MIL backbone prior to slide-level aggregation. For each tile *i*, the backbone maps the encoder feature x_*i*_ to an embedding **h**_*i*_ = *f*_*θ*_(**x**_i_) and produces a raw attention score *a*_*i*_ (i.e., the logit before “softmax”). Attention weights are obtained via slide-wise softmax over the *N* tiles in the slide, followed by a rescaling step:

$${\tilde{\alpha }}_{i}=\frac{\;\exp\;\left(a_{i}\right)}{\sum_{j=1}^{N}\;\exp\;\left(a_{j}\right)},\alpha_{i}=N\cdot {\tilde{\alpha }}_{i}\text{.}$$

${\tilde{\alpha }}_{i}$ denotes the normalized attention weight and *N* denotes the number of tiles extracted from the current slide. The rescaling sets the mean weight within each slide to 1 (since $\frac{1}{N}\sum_{i=1}^{N}\alpha_{i}=1$), which makes the average magnitude of attention weights comparable across slides with different numbers of tiles while preserving the relative ranking of tiles within each slide. The resulting attention-structured *h*-space representation of a slide is given by

$$H={\left\{\left(h_{i},\alpha_{i}\right)\right\}}_{i=1}^{N}\text{.}$$

This standardized *h*-space representation above serves as the input to CLEAR-HPV’s unsupervised concept-discovery procedure, which analyzes the AW distribution of tile embeddings to reveal structured morphologic patterns learned by the MIL model.

#### Concept-discovery module

CLEAR-HPV performs unsupervised concept discovery by clustering the attention-structured *h*-space representation into a fixed set of *K* latent morphologic concepts, without requiring any concept-level annotations (labels). In Table 2, we compare CLEAR-HPV (AW-*h*) and CLEAR-HPV (raw-*h*) against several unsupervised baselines, including heatmap, Dirichlet concepts, and encoder concepts. We describe each method in detail below. All methods operate on tile-level *h*-space representation {(**h**_*i*_,*α*_*i*_)} and are designed to scale to whole-slide inference.

#### CLEAR-HPV (raw-*h*)

The raw variant of CLEAR-HPV, i.e., CLEAR-HPV (raw-*h*), discovers *K* morphologic concepts by clustering tile embeddings in the *h*-space using a scalable two-stage *k*-means procedure.^38^^,^^39^ Let ${\left\{h_{i}\right\}}_{i=1}^{N}$ denote the *N* tile embeddings used for concept learning, where each $h_{i}\in {\mathbb{R}}^{d_{h}}$ is a *d*_*h*_-dimensional feature vector for tile *i*. We learn *K* concept centroids ${\left\{\mu_{k}\right\}}_{k=1}^{K}$ with $\mu_{c}\in {\mathbb{R}}^{d_{h}}$ and a hard assignment function *c*(*i*)∈{1, …,*K*} that maps each tile *i* to its nearest centroid by minimizing the within-cluster sum of squared distances:

(Equation 1) $${\left\{\mu_{k}\right\}}_{k=1}^{K},{\left\{c\left(i\right)\right\}}_{i=1}^{N}=\underset{\left\{\mu_{k}\right\},c\left(i\right)}{\mathrm{argmin}}\sum_{i=1}^{N}{\left\|h_{i}-\mu_{c\left(i\right)}\right\|}_{2}^{2}\text{.}$$

In practice, we initialize concept centroids in the *d*_*h*_-dimensional *h*-space using a reservoir-based mini-batch *k*-means pass, followed by standard *k*-means clustering^40^ applied to all tile embeddings to obtain stable concept centroids. This variant treats all tiles equally during concept learning. We next introduce CLEAR-HPV (AW-*h*), which incorporates attention weights when forming concepts and provides a complementary view of the attention-structured *h*-space.

#### CLEAR-HPV (AW-*h*)

To incorporate the MIL model’s diagnostic relevance into concept learning, CLEAR-HPV (AW-*h*) uses an AW *k*-means objective. Let ${\left\{h_{i}\right\}}_{i=1}^{N}$ denote tile embeddings and let *α*_*i*_ ≥ 0 denote the corresponding attention weight assigned by the MIL model (with $\sum_{i}\alpha_{i}>0$). We learn *K* centroids ${\left\{\mu_{k}\right\}}_{k=1}^{K}$ and hard assignments *c*(*i*)∈{1, …,*K*} by solving

(Equation 2) $${\left\{\mu_{k}\right\}}_{k=1}^{K},{\left\{c\left(i\right)\right\}}_{i=1}^{N}=\underset{\left\{\mu_{k}\right\},c\left(i\right)}{\mathrm{argmin}}\sum_{i=1}^{N}\alpha_{i}{\left\|h_{i}-\mu_{c\left(i\right)}\right\|}_{2}^{2}\text{.}$$

During initialization and refinement, tile weights *α*_*i*_ act as sample weights, causing high-attention tiles to exert a stronger influence on concept boundaries. This aligns the learned concepts with regions emphasized by the MIL model.

#### Dirichlet concepts

Building on ideas from probabilistic concept models such as PACE,^41^ we include a probabilistic concept model that learns the uncertainty-aware importance score for each tile and each concept for the prediction. A probabilistic model like this is usually more robust and captures broader variability in morphologic patterns across slides.

#### Heatmap

We evaluated a heatmap-based slide classifier that uses the MIL attention map as a quantitative decision signal. For each slide, tile-level attention scores are projected back onto the WSI to form a spatial heatmap. A slide-level prediction is obtained by computing a scalar “heatmap area” score, defined as the proportion of tiles whose attention exceeds a fixed threshold *τ* for the class of interest. The decision cutoff is selected on training data and applied to held-out test slides, yielding a transparent rule-based classifier grounded in attention-based evidence.

#### Encoder concepts

As a baseline, we evaluate an encoder-based concept-discovery method that follows the same procedure as CLEAR-HPV (raw-*h*) but operates directly on the encoder feature space rather than the MIL latent *h*-space. Let ${\left\{z_{i}\right\}}_{i=1}^{N}$, with $z_{i}\in {\mathbb{R}}^{d_{e}}$, denote the tile-level embeddings produced by the pretrained encoder; all subsequent steps are identical to CLEAR-HPV (raw-*h*). Because this baseline does not involve MIL attention, an AW (AW-*h*) variant is not applicable.

#### Choice of number of concepts (*K*)

We use *K* = 10 as the main setting in our experiments. This choice was initially guided by elbow analysis^42^ and further supported by evaluations across nearby values of *K*. These analyses show that predictive performance remains stable and that the overall structure of the discovered concepts is highly consistent across different values of *K*. In practice, changing *K* mainly merges or splits similar concepts rather than producing entirely different patterns. We therefore do not interpret *K* = 10 as a biologically unique optimum but as a stable and interpretable choice for this study.

### Concept-fraction vectors in CLEAR-HPV

As shown in Figures 1D and 1G, our CLEAR-HPV provides both class-level and slide-level concept-fraction vectors to enable holistic interpretation (explanation) of MIL models’ predictions. Below, we describe details on how they are computed.

#### Raw slide-level concept-fraction vectors

Given a set of *K* discovered concepts from CLEAR-HPV, we represent each WSI with a concept-fraction vector that summarizes its morphologic composition. Specifically, for a slide with *N* tiles, let *c*(*i*) ∈ {1, …,*K*} denote the index of the concept assigned to tile *I* ∈ {1,2, …,*N*}. The slide-level concept-fraction vector $f\in {\mathbb{R}}^{K}$ is defined as

(Equation 3) $$f_{k}=\frac{1}{N}\sum_{i=1}^{N}I\left[c\left(i\right)=k\right],k=1,\ldots ,K\text{,}$$

where *f*_*k*_ is the *k*-th entry in **f** and I[·] is the indicator function, equal to 1 if tile *i* is assigned to concept *k* and 0 otherwise. Each entry *f*_*k*_ therefore represents the proportion of tiles in the slide assigned to concept *k*, and the vector **f** is normalized such that $\sum_{k=1}^{K}f_{k}=1$.

#### AW slide-level concept-fraction vectors

For AW variants, each tile *i* is also associated with an attention weight *α*_*i*_ produced by the MIL model, reflecting its contribution to the slide-level prediction. Correspondingly, the AW concept fraction vectors are computed as

(Equation 4) $$f_{k}=\frac{\sum_{i=1}^{N}\alpha_{i}\;I\left[c\left(i\right)=k\right]}{\sum_{i=1}^{N}\alpha_{i}}\text{,}$$

where tiles receiving higher attention contribute more strongly to the slide-level representation. Attention weights are normalized within each slide to ensure comparability across slides of different sizes.

These two variants of slide-level concept-fraction vectors are used as inputs to the concept-fraction classifier for quantitative evaluation.

#### Class-averaged concept-fraction vectors

For interpretability analyses, we further compute class-averaged concept-fraction vectors by averaging slide-level concept-fraction vectors across all slides in a given cohort or clinical group (e.g., averaging over HPV-positive or HPV-negative cases). Throughout the paper, we use the term concept-fraction vector to denote slide-level representations and class-averaged concept-fraction vectors when these representations are averaged within a subgroup of a cohort (e.g., HPV-positive cases) for interpretability analyses.

### Representative tiles per concept

As shown in Figure 1E, to support qualitative interpretation of the discovered concepts, we identify representative tiles for each concept using an automated and reproducible ranking procedure. Tiles are ranked by their Euclidean distance to the corresponding concept centroid $\mu_{k}\in {\left\{\mu_{k}\right\}}_{k=1}^{K}$, as defined by Equation 1, and the top *M* tiles are selected across slides for visualization. These representative tiles are used exclusively for qualitative interpretation and do not influence model training or evaluation.

### Spatial concept maps

#### Spatial concept maps

As shown in Figures 1F and 5A(ii), to visualize the spatial organization of discovered concepts within WSIs, we generate spatial concept maps by projecting tile-level concept assignments back to their original spatial locations in the WSI. Each tile is assigned to a single concept using hard assignment, and tiles are colored according to their assigned concept, producing a map that illustrates how different morphologic concepts are distributed across the tissue section.

#### High-attention spatial concept maps

As shown in Figure 5A(iii), when attention scores are available, we additionally generate a high-attention map by displaying only the top *M* tiles ranked by MIL attention score, highlighting regions most strongly emphasized by the model.

### Evaluation of concept-fraction vectors

All concept-discovery methods were evaluated under a consistent 10-fold train/test protocol applied to the *h*-space. For each fold, concepts were learned using only training slides, and concept-fraction vectors were computed for both training and held-out slides. Slide-level predictions derived from concept fractions were compared against the baseline MIL classifier.

#### Concept-fraction classifier

Given a slide S, let $f\left(S\right)=\left[f_{1},\ldots ,f_{K}\right]\in {\mathbb{R}}^{K}$ denote its slide-level concept-fraction vector as defined in Equations 3 and 4, where *f*_*k*_ is the proportion of tiles assigned to concept *k* and $\sum_{k=1}^{K}f_{k}=1$. We then define a simple, interpretable rule-based classifier that scores each slide by aggregating the fractions of concepts that are statistically associated with HPV-positive status in the training data. Specifically, for each concept *k*, we compare the distribution of *f*_*k*_ between HPV-positive and HPV-negative training slides and designate concept *k* as HPV associated if its mean fraction is higher in the HPV-positive group. This yields a binary concept mask ***π*** ∈ {0,1}^*K*^, where *π*_*k*_ = 1 indicates that concept *k* is positively associated with HPV status based on the training set, and *π*_*k*_ = 0 otherwise.

The slide-level score is computed as

$$s_{\text{rule}}\left(S\right)=\sum_{k=1}^{K}\pi_{k}\;f_{k}\left(S\right)\text{,}$$

which represents the total fraction of tissue assigned to HPV-associated concepts. A binary prediction is obtained by thresholding this score,

$$\hat{y}\left(S\right)=I\left[s_{\text{rule}}\left(S\right)\geq \tau \right]\text{,}$$

where $\hat{y}\left(S\right)=1$ denotes an HPV-positive prediction and $\hat{y}\left(S\right)=0$ denotes an HPV-negative prediction. The decision threshold *τ* is selected using the training folds and then applied to held-out test slides.

For comparison, we also train a logistic regression classifier directly using the same concept-fraction vector $f\left(S\right)$ as input. Detailed performance metrics for HPV and survival prediction are reported in Tables S8 and S9.

#### Recovery score

Our CLEAR-HPV is a post hoc explanation framework. It is therefore important to evaluate whether the generated explanation indeed reflects the explained model’s prediction. To measure how well CLEAR-HPV variants and other baselines recover MIL predictions, we computed a recovery score by forming a metric vector,

m=[ACC,AUC,Prec,Rec,Spec,F1],

for each method (e.g., **m**_*CLEAR*-*HPV*_ for CLEAR-HPV and **m**_*Base*_ for a certain base model) and calculating its Euclidean distance *d* to the base model (e.g., CLAM) in the same fold. The final recovery score (for each fold) is

$$s=\frac{1}{1+d},d={\left\|m_{CLEAR-HPV}-m_{Base}\right\|}_{2}\text{.}$$

## Resource availability

### Lead contact

Requests for further information and resources should be directed to and will be fulfilled by the lead contact, Hao Wang (hw488@cs.rutgers.edu).

### Materials availability

This study did not generate new materials.

### Data and code availability

• The histopathology datasets analyzed in this study are publicly available from TCGA and the CPTAC through the Genomic Data Commons portal (https://portal.gdc.cancer.gov) and CPTAC Data Portal (https://proteomics.cancer.gov/data-portal).
• All original code, preprocessing scripts, and trained model checkpoints are available on GitHub (https://github.com/Wang-ML-Lab/CLEAR-HPV) and Zenodo (https://zenodo.org/records/18112878).^43^

## Acknowledgments

H.W. is partially supported by an Amazon Faculty Research Award, Microsoft AI & Society Fellowship, NSF CAREER Award IIS-2340125, NIH grant R01CA297832, and NSF grant IIS-2127918. W.Q. and S.T. are both partially supported by NIH grant R01CA297832.

## Author contributions

Conceptualization, W.Q. and H.W.; investigation, W.Q.; data curation, Y.L.-S.; methodology, W.Q. and H.W.; writing – original draft, W.Q. and H.W.; writing – review & editing, Y.L.-S., H.W., and S.T.; funding acquisition, H.W.; supervision, H.W. and Y.L.-S.

## Declaration of interests

The authors declare no competing interests.

## Declaration of generative AI and AI-assisted technologies in the writing process

During the preparation of this work, the authors used ChatGPT to assist with manuscript editing, code refinement, and literature summarization. After using this tool, the authors reviewed and edited the content as needed and take full responsibility for the content of the publication.

## Supplemental information

Document S1. Figure S1, Tables S1–S9, and Methods S1

Document S2. Article plus supplemental information
